# Kinase-Dead BRAF and Oncogenic RAS Cooperate to Drive Tumor Progression through CRAF

**DOI:** 10.1016/j.cell.2009.12.040

**Published:** 2010-01-22

**Authors:** Sonja J. Heidorn, Carla Milagre, Steven Whittaker, Arnaud Nourry, Ion Niculescu-Duvas, Nathalie Dhomen, Jahan Hussain, Jorge S. Reis-Filho, Caroline J. Springer, Catrin Pritchard, Richard Marais

**Affiliations:** 1 The Institute of Cancer Research, Signal Transduction Team, Section of Cell and Molecular Biology, 237 Fulham Road, London SW3 6JB, UK; 2 The Institute of Cancer Research, Gene and Oncogene Targeting Team, Cancer Research UK Centre for Cancer Therapeutics, 15 Cotswold Road, Sutton, Surrey SM2 5NG, UK; 3 Department of Biochemistry, University of Leicester, University Road, Leicester LE1 7RH, UK; 4 The Institute of Cancer Research, Breakthrough Breast Cancer Research Centre, Fulham Road, London SW3 6JB, UK

**Keywords:** HUMDISEASE, SIGNALING, PROTEINS

## Abstract

We describe a mechanism of tumorigenesis mediated by kinase-dead BRAF in the presence of oncogenic RAS. We show that drugs that selectively inhibit BRAF drive RAS-dependent BRAF binding to CRAF, CRAF activation, and MEK–ERK signaling. This does not occur when oncogenic BRAF is inhibited, demonstrating that BRAF inhibition per se does not drive pathway activation; it only occurs when BRAF is inhibited in the presence of oncogenic RAS. Kinase-dead BRAF mimics the effects of the BRAF-selective drugs and kinase-dead Braf and oncogenic Ras cooperate to induce melanoma in mice. Our data reveal another paradigm of BRAF-mediated signaling that promotes tumor progression. They highlight the importance of understanding pathway signaling in clinical practice and of genotyping tumors prior to administering BRAF-selective drugs, to identify patients who are likely to respond and also to identify patients who may experience adverse effects.

**PaperClip:**

## Graphical abstract

**Graphical Abstract:**

**Figure:**
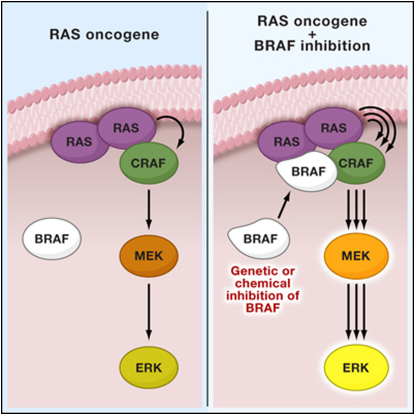

**Highlights:**

► BRAF activates ERK but in some circumstances BRAF inhibitors can induce tumor growth ► BRAF inhibitors drive BRAF-CRAF binding, activating ERK in cells with oncogenic RAS ► Kinase-dead mutants of BRAF have the same effect as BRAF inhibitors ► Oncogenic RAS and kinase-dead BRAF cooperate to induce melanoma in mice

## Introduction

The RAS–ERK (extracellular-signal regulated protein kinase) MAPK (mitogen-activated protein kinase) signaling pathway regulates cell responses to environmental cues (Marshall, 1995) and plays an important role in human cancer (Gray-Schopfer et al., 2007). The pathway comprises the RAS small guanine-nucleotide binding protein and the protein kinases RAF, MEK (mitogen and extracellular-regulated protein kinase kinase), and ERK. RAS is attached to the inner face of the plasma membrane and is activated downstream of growth factor, cytokine, and hormone receptors. Active RAS recruits RAF to the membrane for activation through a complex process involving changes in phosphorylation and binding to other enzymes and scaffold proteins (Kolch, 2000). RAF phosphorylates and activates MEK, which phosphorylates and activates ERK.

The complexity of this pathway is increased by the multiplicity of its components. There are three *RAS* (*HRAS*, *NRAS*, and *KRAS*), three *RAF* (*ARAF*, *BRAF*, and *CRAF*), two *MEK* (*MEK1* and *MEK2*), and two *ERK* (*ERK1* and *ERK2*) genes that encode proteins with nonredundant functions. Furthermore, the pathway is not linear. BRAF binds to and activates CRAF in a RAS-dependent manner that appears to require CRAF transphosphorylation by BRAF (Garnett et al., 2005; Rushworth et al., 2006; Weber et al., 2001), providing subtle pathway regulation that is not fully understood. ERK phosphorylates many substrates and the duration and intensity of its activity affects how cells respond to extracellular signals (Marshall, 1995). Thus, the pathway must be carefully controlled to ensure appropriate responses to environmental cues. In normal cells, outcomes include survival, proliferation, senescence, and differentiation, but in cancer the constitutive pathway activation favors proliferation and survival.

RAS–ERK signaling is particularly important in melanoma. Somatic mutations occur in *BRAF*, *NRAS*, and *KRAS* in 43%, 20%, and 2% of melanomas respectively (www.sanger.ac.uk/genetics/CGP/cosmic/). The mutations in RAS trap it in a GTP-bound, active conformation and mostly involve glycine 12 (G12), glycine 13 (G13), and glutamine 61 (Q61). A glutamic acid substitution for the valine at position 600 (^V600E^BRAF) accounts for over 90% of the mutations in BRAF in cancer. However, over 100 other rare mutations have been described, most of which cluster to the glycine-rich loop and activation segment in the kinase domain. These regions normally trap BRAF in an inactive conformation by forming an atypical intramolecular interaction, and it is thought that the mutations disrupt this interaction, thereby allowing the active conformation to prevail (Wan et al., 2004).

Functional studies have shown that most of the mutations in BRAF are activating and enhance its ability to directly phosphorylate MEK (Wan et al., 2004; Garnett and Marais, 2004). Curiously however, some mutants have impaired activity and although they cannot directly phosphorylate MEK, they appear to retain sufficient activity to bind to and transphosphorylate and activate CRAF in a RAS-independent manner (Garnett et al., 2005), allowing these mutants to activate the pathway indirectly through CRAF. More puzzling are mutations that occur at aspartic acid 594 (D594). The carboxy oxygen of this highly conserved residue (the “D” of the DFG motif) plays a critical role in chelating Mg^2+^ and stabilizing ATP binding in the catalytic site (Johnson et al., 1998). As in other kinases, mutation of this residue causes inactivation and thus cancer mutants such as ^D594V^BRAF cannot phosphorylate MEK, activate CRAF, or stimulate cell signaling (Ikenoue et al., 2003; Wan et al., 2004). These mutants therefore appear catalytically and biologically inactive and yet 34 have been found in human cancer (www.sanger.ac.uk/genetics/CGP/cosmic/). Furthermore, while ^V600E^BRAF mutations (over 10,000 described) occur in a mutually exclusive manner with RAS mutations, four of the 34 kinase-dead mutants are coincident with RAS mutations, a highly significant enrichment (p < 10^−9^; Fisher's Exact Test) that suggests functional interaction.

It has been shown that ^V600E^BRAF is 500-fold activated, can stimulates constitutive MEK–ERK signaling in cells (Gray-Schopfer et al., 2007) and induce melanoma in mice (Dankort et al., 2009; Dhomen et al., 2009), showing that it can be a founder mutation in melanoma. Importantly, ^V600E^BRAF inhibition blocks melanoma cell proliferation and induces apoptosis in vitro and blocks melanoma xenograft growth in vivo (see Gray-Schopfer et al., 2007). These data validate ^V600E^BRAF as a driver of melanomagenesis and as a therapeutic target in melanoma, so drugs to target this pathway have been developed. The first to be tested clinically were the multi-kinase inhibitor sorafenib and the MEK inhibitor PD184352 (CI1040). Disappointingly, both failed to produce objective responses in patients, either because they were not sufficiently potent, or because they caused unacceptable toxicity (Halilovic and Solit, 2008). Recently, more potent and selective BRAF inhibitors have been described. For example, the triarylimidazole SB590885 and the difluorophenylsulfonamine PLX4720 display excellent selectivity for BRAF in vitro and preferentially inhibit BRAF mutant cancer cell proliferation (King et al., 2006; Tsai et al., 2008). More importantly, BRAF-selective drugs have recently entered the clinic and are producing excellent responses in patients with BRAF mutant melanoma (Flaherty et al., 2009; Schwartz et al., 2009).

The aim of this study was to better understand the responses that melanoma cells make to BRAF-selective inhibitors and thereby to provide a molecular basis for the design of clinical trials using BRAF drugs. We also wished to examine if kinase-dead BRAF and oncogenic RAS functionally interact in vivo.

## Results

### BRAF Inhibitors Activate MEK and ERK in RAS Mutant Melanoma Cells

**Figure S1:**
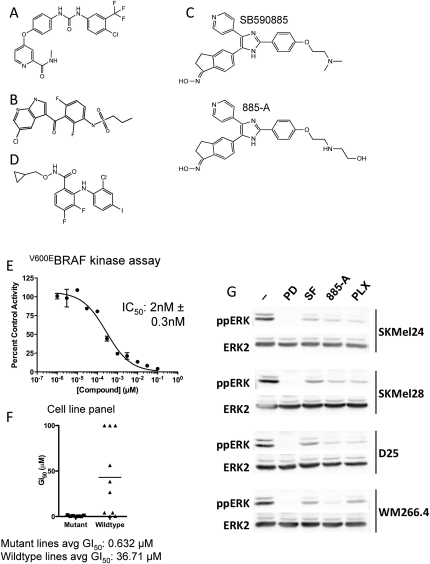
Characterization of 885-A, an Analog of SB590885, Relates to Figure 1 (A) Sorafenib, a pan-RAF, multi-kinase class II inhibitor. (B) PLX4720, a selective class I BRAF inhibitor. (C) SB590885 and its analog 885-A, selective class I BRAF inhibitors. (D) PD184352, a MEK inhibitor (CI1040). (E) Inhibition of ^V600E^BRAF by 885-A in vitro. Insect cell purified ^V600E^BRAF kinase activity was measured at increasing concentrations of 885-A using a 96-well DELFIA-based assay system (Perkin Elmer, Amersham, UK). The assays were performed in the linear range of the assay and in duplicate using a concentration response of 11 points. IC_50_ values were determined using GraphPad Prism software (GraphPad Software, San Diego, CA) and the reported IC_50_ values are the mean of 3 independent assays. (F) 885-A selectively inhibits the growth of BRAF mutant melanoma cells. The growth inhibitory activity of 885-A against a panel of cell lines (7 V600 BRAF mutant lines; 10 wild-type BRAF lines) is represented. The individual IC_50_ values for each line are represented by the symbols, and the horizontal line represents the mean IC_50_ for these two populations. (G) SKMel24, SKMel28, D25 and WM266.4 cells were treated with DMSO (-), PD184352 (PD; 1μM), sorafenib (SF; 10 μM), 885-A (1μM) and PLX4720 (PLX; 0.3μM) for 4 hr. Cell extracts were western blotted for phospho-ERK (ppERK) and total ERK2 (loading control).

We selected four drugs for our studies (Figures S1A–S1D). Sorafenib is a class II (inactive conformation binder) drug (Wan et al., 2004) that inhibits ^V600E^BRAF at 40 nM, CRAF at 13 nM, and several other kinases in the low nM range (Wilhelm et al., 2004). It is the least-selective drug that we used. PLX4720 is a class I (active conformation binder) inhibitor that is highly selective and inhibits ^V600E^BRAF at 13 nM (Tsai et al., 2008). 885-A (Figure S1C) is a close analog of the class I inhibitor SB590885 (King et al., 2006) that is also highly selective for BRAF. It inhibits ^V600E^BRAF at 2 nM (Figure S1E), is ineffective against a panel of 64 other protein kinases (Table S1), and preferentially blocks BRAF mutant cancer cell proliferation (Figure S1F). Finally, we also used the potent and selective MEK inhibitor PD184352 (Sebolt-Leopold et al., 1999).

**Figure 1:**
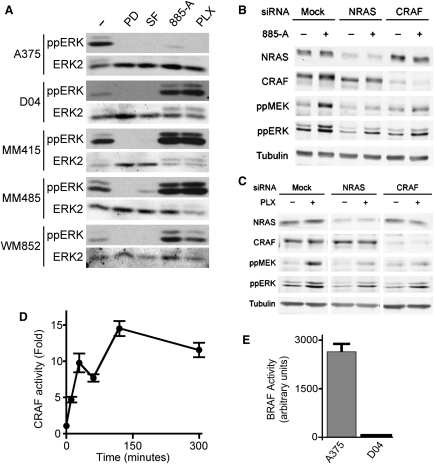
BRAF Inhibitors Activate CRAF, MEK, and ERK in RAS Mutant Cell Lines (A) A375, D04, MM415, MM485, and WM852 cells were treated with DMSO (−), PD184352 (PD; 1 μM), sorafenib (SF; 10 μM), 885-A (1 μM) and PLX4720 (PLX; 0.3 μM) for 4 hr. Cell extracts were western blotted for phospho-ERK (ppERK) and total ERK2 (loading control). (B and C) D04 cells were transfected with siRNA against NRAS or CRAF, or control (Mock) as indicated. After 48 hr the cells were treated with DMSO (−), 885-A (1 μM) or PLX4720 (PLX; 0.3 μM) for 4 hr. Cell lysates were western blotted for NRAS, CRAF, phospho-MEK (ppMEK), phospho-ERK (ppERK) and tubulin (loading control). (D) D04 cells were treated with 885-A for various times and endogenous CRAF kinase activity was measured. Data show fold activation of experimental triplicates compared to untreated cells with error bars to represent standard deviations from the means. (E) Endogenous BRAF kinase activity was measured in A375 or D04 cells. The results (arbitrary units per μg of cell protein) are the mean of an assay performed in triplicate with error bars to represent standard deviation from the mean.

As expected, all four drugs blocked ERK activity in BRAF mutant A375 melanoma cells (Figure 1A; see Table S2). Similarly, all four drugs inhibited ERK in SkMel24, SkMel28, D25, and WM266.4 cells, another four lines that express mutant BRAF (Figure S1G). We also tested the drugs in D04, MM415, MM485, and WM852 NRAS mutant cells (Table S2). As expected, PD184352 and sorafenib inhibited ERK in all of these lines (Figure 1A). Surprisingly, however, PLX4720 and 885-A caused an unexpected increase in ERK activity in the NRAS mutant cells (Figure 1A). NRAS or CRAF depletion by RNA interference (RNAi) blocked MEK/ERK activation by PLX4720 and 885-A in NRAS mutant cells (Figure 1B and 1C) and we show that 885-A activated CRAF in these cells (Figure 1D). We previously reported that oncogenic RAS requires CRAF but not BRAF to activate MEK (Dumaz et al., 2006) and consistent with this, BRAF is inactive in NRAS mutant cells (Figure 1E). These data therefore present an intriguing paradox. BRAF is not active and is not required for MEK/ERK activation in RAS mutant cells. Nevertheless, BRAF inhibitors hyperactivate CRAF and MEK in these cells, so we studied the underlying mechanism(s).

### RAF Inhibitors Induce BRAF Binding to CRAF in RAS Mutant Cells

**Figure 2:**
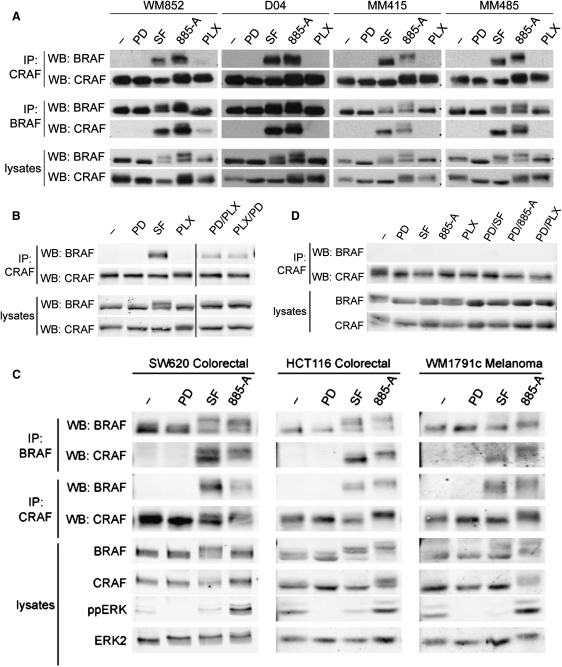
BRAF Inhibitors Induce CRAF Binding to BRAF (A) WM852, D04, MM415 and MM485 cells were treated with DMSO (−), PD184352 (PD; 1 μM), sorafenib (SF; 10 μM), 885-A (1 μM) or PLX4720 (PLX; 0.3 μM) for 4 hr. Endogenous BRAF (IP: BRAF) or endogenous CRAF (IP: CRAF) were immunoprecipitated and the immunocomplexes were western blotted (WB) for BRAF or CRAF. BRAF, and CRAF levels in the cell lysates are also shown. (B) D04 cells were treated with DMSO (−), PD184352 (PD; 1 μM), sorafenib (SF; 10 μM) and PLX4720 (PLX; 0.3μM) for 4 hr. Endogenous CRAF (IP: CRAF) was immunoprecipitated and the immunocomplexes were western blotted (WB) for BRAF or CRAF. BRAF and CRAF levels in the cell lysates are shown. (C) SW620, HCT116 and WM1791c cells were treated with DMSO (−), PD184352 (PD; 1 μM), sorafenib (SF; 10 μM) or 885-A (1 μM) for 4 hr. Endogenous BRAF (IP: BRAF) or endogenous CRAF (IP: CRAF) were immunoprecipitated and the immunocomplexes were western blotted (WB) for BRAF or CRAF. The cell lysates were also blotted for BRAF, CRAF, phospho-ERK (ppERK) and total ERK2 (loading control). (D) A375 cells were treated with DMSO (−), PD184352 (PD; 1 μM), sorafenib (SF; 10 μM), 885-A (1 μM) or PLX4720 (PLX; 0.3 μM) for 4 hr. CRAF (IP: CRAF) was immunoprecipitated and the immunocomplexes were western blotted (WB) for BRAF or CRAF. BRAF and CRAF levels in the cell lysates are shown.

**Figure S2:**
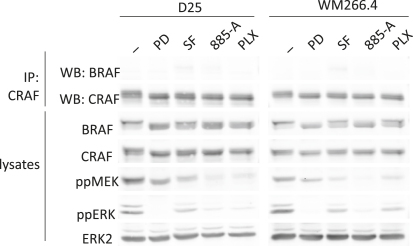
MEK, pan-RAF or BRAF Inhibitors Do Not Induce Strong BRAF Binding to CRAF in BRAF Mutant Melanoma Cell Lines, Relates to Figure 2 D25 and WM266.4 cells were treated with DMSO (-), PD184352 (PD; 1μM), sorafenib (SF; 10 μM), 885-A (1μM) or PLX4720 (PLX; 0.3μM) for 4 hr. Endogenous CRAF (IP: CRAF) was immunoprecipitated and the immunocomplexes were western blotted (WB) for BRAF and CRAF. BRAF, CRAF, phospho-MEK (ppMEK), phospho-ERK (ppERK) and total ERK2 levels in the cell lysates are also shown.

Wild-type BRAF binds to CRAF in a RAS-dependent manner and although this binding is weak, it leads to CRAF activation (Garnett et al., 2005). Since RAS and CRAF are required for ERK activation by PLX4720 and 885-A, we investigated if these drugs induce BRAF binding to CRAF. Endogenous BRAF was immunoprecipitated from melanoma cells and western blotted for endogenous CRAF. We show that CRAF did not bind to BRAF in untreated or PD184352 treated WM852, D04, MM415, or MM485 cells (Figure 2A), demonstrating that MEK inhibition does not induce binding. In contrast, sorafenib and 885-A induced strong binding of BRAF to CRAF in all four lines (Figure 2A). We also performed the experiment in the inverse manner, immunoprecipitating CRAF and showing that BRAF binding was strongly induced by sorafenib and 885-A (Figure 2A). Curiously, PLX4720 did not appear to induce BRAF binding to CRAF, but previous studies have shown that ERK phosphorylates BRAF in a negative-feedback loop that destabilizes its binding to CRAF (Rushworth et al., 2006). We show that PD184352 stabilizes BRAF binding to CRAF in the presence of PLX4720 (Figure 2B), demonstrating that PLX4720 does induce binding, albeit less strongly than the other drugs. In addition to inducing BRAF binding to CRAF in NRAS mutant cells, 885-A and sorafenib also induce this binding in WM1791c melanoma cells and in SW620 and HCT116 colorectal carcinoma cells (Figure 2C), all of which express mutant *KRAS* (Table S2). Importantly, no strong binding of BRAF to CRAF was seen in A375 cells even in the presence of PD184352 and the drugs did not induce strong BRAF binding to CRAF in two other BRAF mutant melanoma cell lines (Figure 2D and Figure S2).

### BRAF Binding to CRAF Is Mediated by RAS

**Figure 3:**
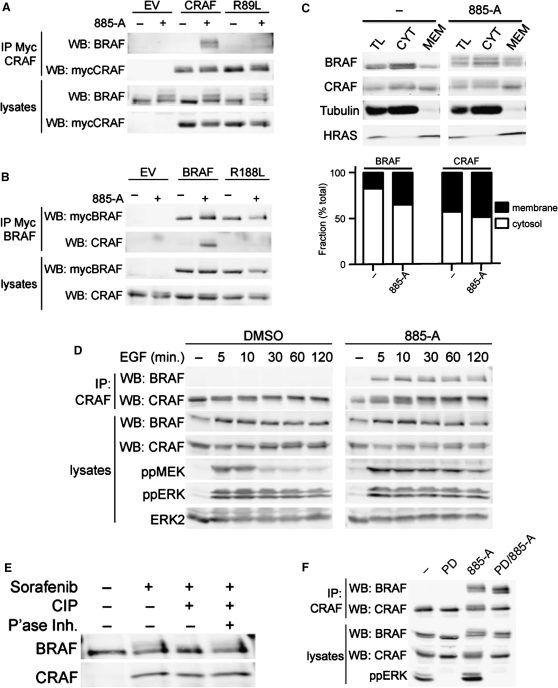
BRAF Binding to CRAF Requires RAS (A) Myc-epitope tagged CRAF or ^R89L^CRAF (R89L), or an empty vector control (EV) were transfected into D04 cells. After 48 hr, the cells were treated with DMSO (−) or 885-A (1 μM) for 4 hr. Myc-tagged CRAF was immunoprecipitated (IP) and the immunocomplexes were western blotted (WB) for endogenous BRAF or myc-CRAF. Endogenous BRAF and myc-CRAF levels in the cell lysates are also shown. (B) Myc-epitope tagged BRAF or ^R188L^BRAF (R188L) or an empty vector control (EV) were transfected into D04 cells. After 48 hr the cells were treated with DMSO (−) or 885-A (1 μM) for 4 hr. Myc-tagged BRAF was immunoprecipitated (IP) and the immunocomplexes were western blotted (WB) for myc-BRAF or endogenous CRAF. Myc-BRAF and endogenous-CRAF levels in the cell lysates are also shown. (C) Membrane or cytosol fractions were prepared from untreated (−) or 885-A (1 μM) treated D04 cells. BRAF, CRAF, Tubulin (cytosol control) and HRAS (membrane control) were western blotted in the total lysate (TL), cytosolic fraction (CYT) and membrane fraction (MEM). The graph shows the quantification of the relative levels of BRAF and CRAF in the membrane and cytosol fractions. (D) PMWK cells were pretreated with DMSO or 885-A (1 μM, 60 min) and then treated with EGF (10 ng/ml) for the times shown in minutes (min). Endogenous CRAF was immunoprecipitated (IP) and the precipitates were western blotted (WB) for BRAF and CRAF. The lysates were also western blotted for BRAF, CRAF, phospho-MEK (ppMEK), phospho-ERK (ppERK) and total ERK2. (E) D04 cells were treated with DMSO (−) or sorafenib (+; 10 μM) for 4 hr. Endogenous BRAF was immunoprecipitated and the immunocomplexes left untreated or incubated with calf intestinal phosphatase (CIP; 5U, 30°C, 30 min) in the presence or absence of phosphatase inhibitors (P'ase Inh). Immunocomplexes were western blotted for BRAF and CRAF. (F) D04 cells were treated with DMSO (−), PD184352 (PD; 1 μM) or 885-A (1 μM) for 4 hr. Endogenous CRAF (IP: CRAF) was immunoprecipitated and the immunocomplexes were western blotted (WB) for BRAF or CRAF. BRAF, CRAF, and phospho-ERK (ppERK) levels in the cell lysates are shown.

**Figure S3:**
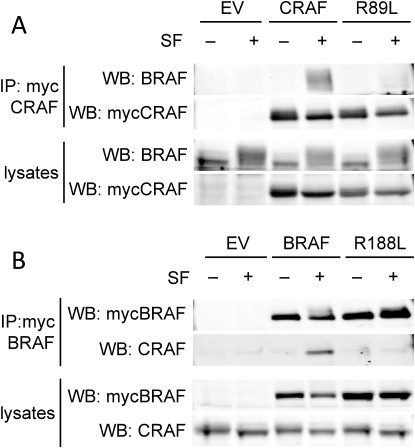
BRAF Binding to CRAF Requires BRAF and CRAF Binding to RAS, Relates to Figure 3 (A) Myc-epitope tagged CRAF or ^R89L^CRAF (R89L), or an empty vector control (EV) were transfected into D04 cells. After 48 hr the cells were treated with DMSO (-) or sorafenib (SF, +; 10 μM) for 4 hr. The myc-CRAF was immunoprecipitated (IP) and the immunocomplexes were western blotted (WB) for endogenous BRAF or myc-CRAF. Endogenous BRAF and myc-CRAF levels in the cell lysates are also shown. (B) Myc-epitope tagged BRAF or ^R188L^BRAF (R188L) or an empty vector control (EV) were transfected into D04 cells. After 48 hr the cells were treated with DMSO (-) or sorafenib (SF, +; 10 μM) for 4 hr. The myc-BRAF was immunoprecipitated (IP) and the immunocomplexes were western blotted (WB) for myc-BRAF or endogenous CRAF. Myc-BRAF and endogenous-CRAF levels in the cell lysates are also shown.

Thus, sorafenib, 885-A and PLX4720 all induced BRAF binding to CRAF in NRAS or KRAS mutant cells, but not in BRAF mutant cells, showing that BRAF inhibition per se did not induce this binding; it only occurred when BRAF was inhibited in the presence of oncogenic RAS. To confirm the essential role of RAS, we show that a CRAF mutant (^R89L^CRAF) that cannot bind to RAS (Fabian et al., 1994) did not bind to BRAF (Figure 3A and Figure S3A) and the corresponding mutant of BRAF (^R188L^BRAF) did not bind to CRAF (Figure 3B and see Figure S3B). We also prepared membrane/cytosol fractionations of RAS mutant cells and show that under normal conditions over 40% of CRAF is in the membrane, whereas BRAF is largely cytosolic (Figure 3C). Notably, 885-A treatment leads to strong recruitment of BRAF to the membrane fraction, whereas CRAF is only weakly affected (Figure 3C). We also show that under normal conditions, EGF did not induce BRAF binding to CRAF in PMWK cells, a line that is wild-type for *BRAF* and *RAS* (Table S2). However, in the presence of 885-A, EGF induced robust binding of BRAF to CRAF in PMWK cells and this resulted in sustained pathway activation (Figure 3D). This shows that BRAF binding to CRAF is induced in the presence of both oncogenic RAS and activated wild-type RAS.

We note that sorafenib and 885-A induce a mobility shift in BRAF in SDS-gels (Figure 2A). BRAF also undergoes a mobility shift in PLX4720 treated cells in the presence of PD184352 (Figure 2B). This mobility shift is reduced when immunoprecipitated BRAF is treated with calf intestinal alkaline phosphatase (CIP; Figure 3E) and PD184352 pretreatment reduced, but did not ablate the magnitude of the shift induced by 885-A (Figure 3F). Importantly, in vitro CIP treatment and cell pretreatment with PD184352 did not prevent BRAF binding to CRAF (Figures 3E and 3F). Together, these data suggest that the BRAF bound to CRAF is hyperphosphorylated through MEK–ERK-dependent and MEK–ERK-independent mechanisms, but that this phosphorylation is not required for BRAF binding to CRAF.

### BRAF Inhibition Activates CRAF

**Figure 4:**
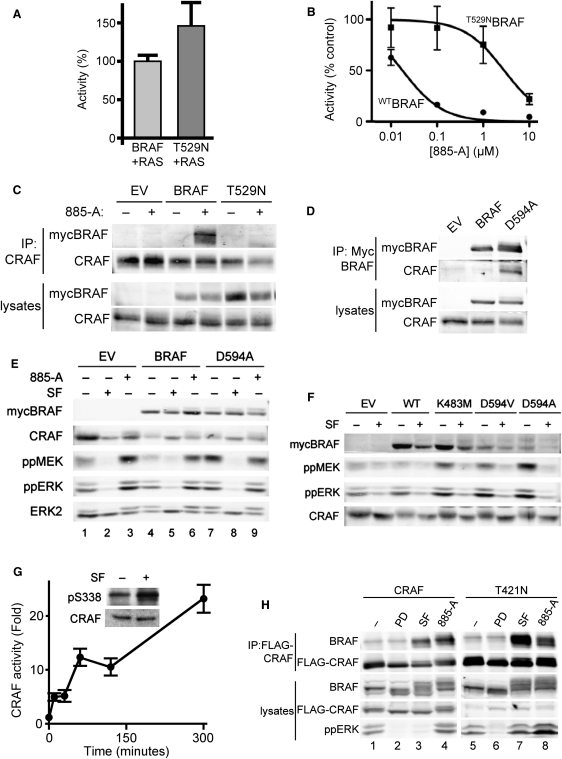
BRAF and Not CRAF Inhibition Drives CRAF Binding to BRAF and CRAF Activation (A) COS cells were transiently transfected with myc-epitope tagged BRAF, or ^T529N^BRAF (T529N) in the presence of ^G12V^HRAS (RAS) and their kinase activity was measured. The data represent one assay performed in triplicate, with error bars to represent standard deviations from the mean. Activity (%) is relative to wild-type BRAF activated by ^G12V^HRAS. (B) As in (A) but immunocomplexes were treated with DMSO (−) or 885-A for 10 min prior to measuring their kinase activity. The data represent one assay performed in triplicate, with error bars to represent standard deviations from the mean. Activity (% control) is relative to the untreated kinase. (C) Myc-epitope tagged BRAF, ^T529N^BRAF (T529N), or an empty vector control (EV) were transfected into D04 cells. After 48 hr the cells were treated with DMSO (−) or 885-A (1 μM) for 4 hr. The endogenous CRAF was immunoprecipitated and the immunocomplexes were western blotted for myc-BRAF or endogenous CRAF. Myc-BRAF and endogenous CRAF levels in the cell lysates are also shown. (D) Myc-epitope tagged BRAF, ^D594A^BRAF (D594A), or an empty vector control (EV) were transfected into D04 cells. After 48 hr the myc-BRAF was immunoprecipitated (IP) and the immunocomplexes were western blotted for mycBRAF and endogenous CRAF. Myc-BRAF and endogenous CRAF levels in the cell lysates are also shown. (E) Myc-epitope tagged BRAF, ^D594A^BRAF (D594A), or an empty vector control (EV) were transfected into D04 cells. After 48 hr the cells were treated with DMSO (−), sorafenib (SF; 10 μM) or 885-A (1 μM) for 4 hr. The cells extracts were western blotted for myc-BRAF, phospho-MEK (ppMEK), phospho-ERK and total ERK2 (loading control). Note that ERK2 runs as a doublet due to the separation of the phosphorylated and nonphosphorylated protein. (F) Myc-epitope tagged BRAF, ^K483M^BRAF (K483M), ^D594V^BRAF (D594V), ^D594A^BRAF (D594A), or an empty vector control (EV) were transfected into D04 cells. After 48 hr, the cells were treated with DMSO (−) or sorafenib (SF; 10 μM) for 4 hr. Cell extracts were blotted for myc-BRAF, phospho-MEK (ppMEK), phospho-ERK (ppERK) and CRAF (loading control). (G) D04 cells were treated with sorafenib (10 μM) for various times and CRAF kinase activity was measured. Data is for one assay performed in triplicate, with error bars to represent standard deviations from the means. Inset: D04 cells were treated with sorafenib (SF) for 4 hr and CRAF was immunoprecipitated and western blotted for S338 phosphorylation (pS338). CRAF levels in the lysate are shown as a loading control. (H) D04 cells stably expressing flag-epitope tagged CRAF (CRAF) or ^T421N^CRAF (T421N) were treated with DMSO (−), PD184352 (PD; 1 μM), sorafenib (SF; 10 μM) or 885-A (1 μM) for 4 hr. The flag-CRAF was immunoprecipitated (IP) and the immunocomplexes were western blotted for endogenous-BRAF or flag-CRAF. Endogenous BRAF, flag-CRAF and phosphorylated ERK (ppERK) levels in the cell lysates are also shown.

**Figure S4:**
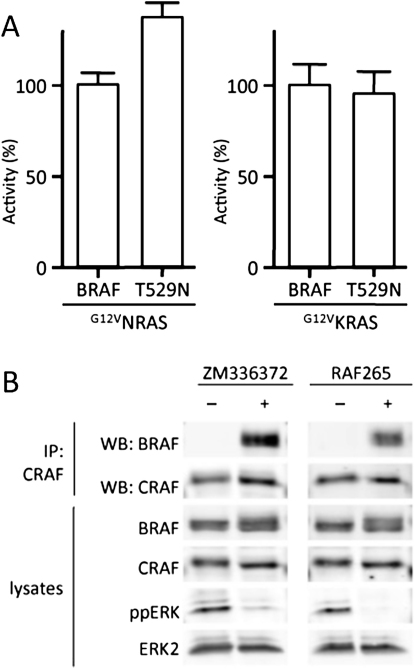
Characterization of ^T529N^BRAF and the pan-RAF Inhibitors ZM336372 and RAF265, Relates to Figure 4 (A) ^T529N^BRAF is activated by NRAS and KRAS. COS cells were transiently transfected with myc-epitope tagged BRAF, or ^T529N^BRAF (T529N) in the presence of ^G12V^NRAS or ^G12V^KRAS as indicated. BRAF kinase activity was measured in an immunoprecipitation kinase assay. The data represent one assay performed in triplicate, with error bars to represent standard deviations from the mean. Activity (%) is relative to wild-type BRAF activated by ^G12V^NRAS or ^G12V^KRAS as appropriate. (B) Pan-RAF inhibitors drive BRAF binding to CRAF but not ERK activation. DO4 cells were treated with ZM336372 or RAF265 for 4 hr. CRAF (IP: CRAF) was immunoprecipitated and the immnocomplexes were western blotted (WB) for BRAF or CRAF. BRAF and CRAF levels in the cell lysates are shown and the lysates were also western blotted for phosphorylated ERK (ppERK) and total ERK2.

To test directly if BRAF binding to CRAF is driven by 885-A binding to BRAF, we mutated the so-called “gatekeeper threonine” (T529) of BRAF to asparagine (T529N). Since BRAF is not active in RAS mutant melanoma cells (Figure 1E), we measured ^T529N^BRAF activity using transient expression in COS cells (Wan et al., 2004). The results show that ^T529N^BRAF is still activated by ^G12V^HRAS, ^G12V^NRAS and ^G12V^KRAS (Figure 4A and Figure S4A). Importantly, ^T529N^BRAF is ∼170-fold less sensitive to 885-A than wild-type BRAF (17 nM versus 2869 nM; Figure 4B) and 885-A did not stimulate its binding to CRAF (Figure 4C), proving that drug binding to BRAF drives BRAF binding to CRAF.

Next, we expressed a kinase-dead version of BRAF (^D594A^BRAF) in D04 cells and show that it forms a constitutive complex with CRAF (Figure 4D) and that it activates MEK constitutively (Figure 4E, compare lanes 1, 4, and 7). Notably, 885-A does not further enhance MEK activation driven by ^D594A^BRAF (Figure 4E, compare lanes 4, 6 to 7, 9), presumably because it cannot further inhibit this already inactive kinase. Two other kinase-dead BRAF mutants, the classical catalytic lysine mutant (^K483M^BRAF), and ^D594V^BRAF, a mutant found in human cancer (Wan et al., 2004), also activate MEK in D04 cells (Figure 4F). Thus, it is BRAF inhibition and not drug binding that drives BRAF binding to CRAF. This experiment also shows that MEK activation driven by kinase-dead BRAF is inhibited by sorafenib (Figures 4E and 4F). Indeed, cell responses to sorafenib appear to be paradoxical. We show that although sorafenib inhibits ERK (Figure 1A), it induces BRAF binding to CRAF (Figure 2A), CRAF activation (Figure 4G) and CRAF phosphorylation on S338 (Figure 4G, inset), a critical event in CRAF activation (Mason et al., 1999). To test directly the role of CRAF in cells when BRAF is inhibited, we mutated its gatekeeper threonine to asparagine (^T421N^CRAF). Notably, ^T421N^CRAF still binds to BRAF in sorafenib and 885-A treated cells (Figure 4H), demonstrating that drug binding to CRAF is not required for BRAF binding to CRAF. More importantly, in the presence of ^T421N^CRAF, sorafenib activates rather than inhibits the pathway (Figure 4H, compare lanes 3 and 7). We therefore posit that sorafenib induces paradoxical activation of CRAF because it inhibits BRAF and drives CRAF activation, but simultaneously binds to and inhibits CRAF. In agreement with this model, we show that two other pan-RAF inhibitors, ZM336372 and RAF265 also induce BRAF binding to CRAF, but without activating ERK (see Figure S4B).

### Oncogenic Ras and Kinase-Dead Braf Cooperate to Induce Melanoma in Mice

**Figure 5:**
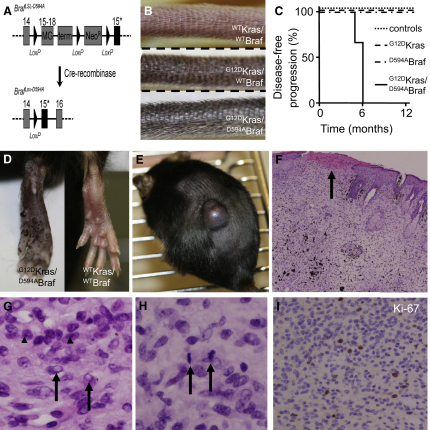
Oncogenic Kras and Kinase-Dead Braf Cooperate to Drive Tumorigenesis (A) Diagrammatic representation of targeted conditional *Braf^LSL-D594A^* allele used for ^D594A^Braf expression in mouse melanocytes. The endogenous mouse *Braf* gene from exons 14–15 is represented. Exon 15 is mutated to express ^D594A^Braf (15^∗^). *LoxP* sites are represented by triangles. The relative position of the wild-type BRAF minigene (MG) comprising exons 15–18 of BRAF, the transcription terminator (term) and the *Neo^R^* cassette are shown. Cre-recombinase mediated removal of these regions results in *Braf^Lox-D594A^*, allowing expression of ^D594A^Braf. (B) Photographs of the tails of tamoxifen-treated wild-type (^WT^Kras/^WT^Braf), *Kras^+/LSL-G12D^*;*Tyr::CreERT2^+/o^* (^G12D^Kras/^WT^Braf), or *Kras^+/LSL-G12D^*;*Braf^+/LSL-D594A^;Tyr::CreERT2^+/o^* (^G12D^Kras/^D594A^Braf) mice. (C) Kaplan-Meier plots showing disease free progression of study mice. The controls consisted of 12 tamoxifen-treated *Tyr::CreERT2^+/o^* mice; 10 ethanol-treated *Braf^+/LSL-D594A^*;*Tyr::CreERT2^+/o^* mice and 6 ethanol-treated *Kras^+/LSL-G12D^*; *Tyr::CreERT2^+/o^* mice. The experimental groups consisted of 12 tamoxifen-treated *Kras^+/LSL-G12D^*;*Tyr::CreERT2^+/o^* (^G12D^Kras), 24 tamoxifen-treated *Braf^+/LSL-D594A^*;*Tyr::CreERT2^+/o^* (^D594A^Braf) mice, and 3 tamoxifen-treated *Kras^+/LSL-G12D^*;*Braf^+/LSL-D594A^;Tyr::CreERT2^+/o^* (^G12D^Kras/^D594A^Braf) mice. (D) Photographs of the feet of tamoxifen-treated wild-type (^WT^Kras/^WT^Braf), or *Kras^+/LSL-G12D^*;*Braf^+/LSL-D594A^;Tyr::CreERT2^+/o^* (^G12D^Kras/^D594A^Braf) mice. (E) Photograph showing a large tumor on the back of a tamoxifen-treated *Kras^+/LSL-G12D^*;*Braf^+/LSL-D594A^;Tyr::CreERT2^+/o^* (^G12D^Kras/^D594A^Braf) mouse. The fur was removed to reveal the lesion. (F) Photomicrograph of a tumor from the back of a ^G12D^Kras/^D594A^Braf mouse. An area of ulceration is highlighted by the arrow. (G) High magnification photomicrograph of a section of tumor showing atypical cells, conspicuous nucleoli (arrowheads) and nuclear pseudo-inclusions (arrows). (H) High magnification photomicrograph of a section of tumor showing mitotic figures (arrows). (I) Photomicrograph of a section of tumor subjected to immunohistochemical analysis with antibodies against Ki67 (MIB1).

Our data establish that inhibition of BRAF in the presence of oncogenic RAS hyperactivates CRAF, MEK, and ERK. To investigate the consequences of this in vivo, we used conditionally targeted alleles of oncogenic Kras (*Kras^LSL-G12D^*) and kinase-dead Braf (*Braf^LSL-D594A^*) in transgenic mice. These alleles use Cre-recombinase/*LoxP*-Stop-*LoxP* (LSL) technology to regulate inducible expression of mutant proteins from the endogenous mouse genes to ensure normal levels of protein expression. The *Kras^LSL-G12D^* allele has been described (Jackson et al., 2001), and we recently developed the *Braf^LSL-D594A^* allele. Briefly, exon 15 of endogenous *Braf* was targeted to mutate D594 to alanine (D594A; see Figure 5A). To prevent expression of ^D594A^Braf in all cells, an LSL cassette was inserted between exon 14 and the mutated exon 15. This contains a minigene for exons 15–18 of ^WT^Braf, a transcription terminator and a *Neo^R^* selection marker to ensure that only ^WT^Braf is expressed. Removal of the LSL cassette by Cre-recombinase reveals the mutated exon 15 and ^D594A^Braf is expressed. These mice were crossed to *Tyr::CreERT2* mice (Yajima et al., 2006), in which the tyrosinase promoter is used to express tamoxifen-activated Cre-recombinase (CreERT2) in the melanocytes. Since CreERT2 is activated by tamoxifen, this approach provides exquisite spatial and temporal control over ^G12D^Kras and ^D594A^Braf expression.

*Kras^+/LSL-G12D^*, *Braf^+/LSL-D594A^*, and *Tyr::CreERT2^+/o^* mice were crossed to generate *Kras^+/LSL-G12D^*;*Tyr::CreERT2^+/o^*, *Braf^+/LSL-D594A^*;*Tyr::CreERT2^+/o^*, or *Kras^+/LSL-G12D^*;*Braf^+/LSL-D594A^*;*Tyr::CreERT2^+/o^* mice. In all cases, the conditionally targeted alleles were balanced over a corresponding wild-type allele. Mice were treated with tamoxifen at 2–3 months of age to induce mutant protein expression. We have recently shown that in this model, ^V600E^Braf induces skin hyperpigmentation, nevus formation, and melanoma (Dhomen et al., 2009). In contrast, ^D594A^Braf did not induce skin hyperpigmentation, nevi (data not shown) or tumors (Figure 5C). ^G12D^Kras induced weak tail darkening after 5–6 months (Figure 5B) but did not induce either nevi (data not shown) or tumors (Figure 5C). However, when ^D594A^Braf and ^G12D^Kras were combined, they induced a conspicuous skin phenotype. Within 2–3 months the ears (data not shown), tails (Figure 5B), and paws (Figure 5D) darkened visibly. The mice did not develop nevi, but within 6 months, they all developed large, rapidly growing oligo-pigmented tumors (Figures 5C and 5E). The tumors displayed evidence of ulceration (Figure 5F) and were composed largely of spindle cells that exhibit features of malignancy, including cellular atypia, nuclear pleomorphism, and conspicuous nucleoli (Figure 5G). They were highly proliferative as evidenced by large numbers of mitotic figures in the superficial and deep aspects of the lesions (∼6 mitosis/10HPF; Figure 5H) and positive staining for Ki67 throughout (Figure 5I).

**Figure 6:**
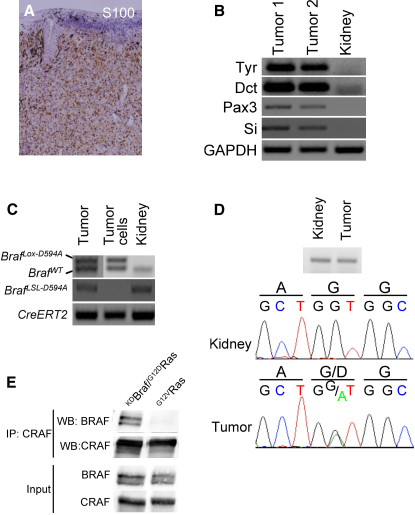
Tumors Induced by Kinase-Dead Braf and Oncogenic Kras Are Melanoma (A) Photomicrograph of a section of tumor subjected to immunohistochemical analysis with antibodies against S100. (B) RT-PCR analysis revealing expression of tyrosinase (Tyr), Dct, Pax3 and silver/gp100 (Si) in two independent tumors and kidney (control). GAPDH is used as a loading control. (C) PCR-mediated genotyping for wild-type Braf (*Braf^WT^*), *Braf^+/LSL-D594A^ and Tyr::CreERT2^+/o^* alleles from a tumor sample, cells derived from the tumor and from kidney as a control. (D) PCR amplified fragment for Kras from kidney and tumor samples. Shown below is the sequencing trace for codons 11–13, together with the DNA and protein sequence (single amino acid code). (E) Endogenous CRAF was immunoprecipitated (IP) from cells derived from a tumor from a ^G12D^Kras/^D594A^Braf mouse (*Kras^+/LSL-G12D^*;*Braf^+/LSL-D594A^;Tyr::CreERT2^+/o^*), or from a tumor from a ^G12V^Kras mouse *(β-actin^+/LSL-G12VKras^;Tyr::CreERT2^+/o^*). The immunocomplexes were western blotted (WB) for Braf and Craf, and the levels of Braf and Craf in the cell lysates are also shown.

The tumors were strongly and diffusely positive for S100 (Figure 6A) and expressed the melanocyte markers tyrosinase, Dct, Pax3, and silver (Figure 6B), consistent with a diagnosis of melanoma. Genomic DNA analysis of the tumors and cell lines derived from them confirmed that *Braf^LSL-D594A^* had been recombined to *Braf^Lox-D594A^* (Figure 6C). However, for technical reasons we could not detect *Kras^LSL-G12D^* recombination (data not shown), so used RT-PCR to amplify and sequence Kras mRNA. We show that only wild-type Kras is expressed in the kidneys, whereas the tumors expressed both wild-type Kras and ^G12D^Kras (Figure 6D). Importantly, we show constitutive binding of Braf to Craf in cells from the ^G12D^Kras/^D594A^Braf tumors (Figure 6E). As a control, we used cells from melanoma induced by ^G12V^Kras overexpression. Briefly, when ^G12V^Kras was overexpressed in melanocytes in mice using the β-actin promoter (β-actin:LSL:^G12V^Kras; Meuwissen et al., 2001), it induced rapid onset melanoma (median time to onset 2 months, 100% penetrance within 3 months) in the absence of ^D594A^Braf (manuscript submitted). Importantly, in cells from these tumors, Braf does not bind to Craf (Figure 6E). Thus, it is only kinase-dead Braf and not wild-type Braf that binds to Craf in the presence of oncogenic Kras.

## Discussion

**Figure 7:**
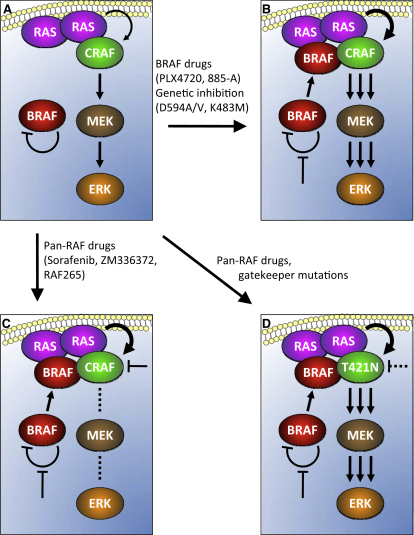
A Model of Paradoxical CRAF Activation by BRAF (A) In the presence of oncogenic RAS, BRAF is cytosolic, where it maintains itself in an inactive conformation in a manner that depends on its own kinase activity. CRAF is recruited to the plasma membrane by RAS and activates the pathway. (B) When BRAF is inhibited by genetic or chemical means, it is no longer autoinhibited and is recruited to the plasma membrane by RAS, where it binds to CRAF. Although BRAF does not itself signal, it can act as a scaffold to enhance CRAF activity and consequently enhance signaling through the pathway. (C) Pan-RAF inhibitors hyperactivate CRAF because they inhibit BRAF, but they simultaneously inhibit CRAF, leading to paradoxical activation of CRAF without pathway activation. (D) ^T421N^CRAF (T421N) escapes the paradoxical activation by the pan-RAF inhibitors, because it no longer allows them to bind, so is freely activated due to BRAF inhibition.

In this study, we show that inhibition of BRAF by chemical or genetic means in the presence of oncogenic or growth-factor activated RAS induces BRAF binding to CRAF, leading to CRAF hyperactivation and consequently elevated MEK and ERK signaling. The mechanism we describe is another paradigm of RAF activation downstream of RAS and based on our findings, we propose the following mechanism by which this occurs. We posit that in RAS mutant cells, BRAF maintains itself in an inactive conformation through its own kinase activity, either through auto-phosphorylation, or by phosphorylating a partner protein that then keeps it inactive (Figure 7A). We are currently using mass-spectrometry and mutagenic approaches to elucidate the underlying mechanism. We propose that when BRAF is inhibited, it escapes this auto-inhibited state and is recruited to the plasma membrane by RAS, where it forms a stable complex with CRAF. Critically, we posit that because it is inhibited, BRAF does not directly phosphorylate MEK, but rather it acts as a scaffold whose function is to enhance CRAF activation, thereby allowing CRAF to hyperactivate the pathway (Figure 7B). We do not know the stoichiometry of the components in these complexes, but since BRAF and CRAF must both bind to RAS for complex formation, it seems likely that at least two RAS proteins are needed to stimulate formation of the complex (Figure 7B).

It is unclear why PLX4720 only induces weak binding of BRAF to CRAF, but this may stem from its unique property of displacing the α-C helix of BRAF when it binds (Tsai et al., 2008) and suggests that this helix is important for BRAF binding to CRAF, something that will only be resolved when the BRAF:CRAF crystal structure is solved. We have attempted to identify other proteins that may be required to stabilize the BRAF–CRAF complexes. Our unpublished mutagenesis data suggests that 14-3-3 is required to stabilize these drug-induced complexes (data not shown) and this is consistent with previous observations demonstrating that 14-3-3 mediates BRAF binding to CRAF (Garnett et al., 2005; Rushworth et al., 2006). Although this appears to contradict our observation that dephosphorylation does not disrupt the complex, because 14-3-3 binds to BRAF and CRAF in a phosphorylation-dependent manner, we presume that 14-3-3 protects these sites from dephosphorylation. We have also used RNAi to examine the potential role of other proteins implicated in BRAF-CRAF complex formation or pathway activation, including the scaffold proteins KSR, Sprouty2 and RKTG and the small G protein RHEB, but our preliminary results have not revealed obvious roles for these proteins. Our studies have parallels to the recently described heterodimers between DRAF and KSR in *Drosophila* (Rajakulendran et al., 2009). Notably, flies have only one RAF isoform and it appears to be an ortholog of BRAF rather than ARAF or CRAF. Our inability to demonstrate an obvious role for KSR in mediating BRAF binding to CRAF or CRAF activation by BRAF suggests that the mechanism underlying dimerization here may be different from those described in flies, but clearly additional studies are required to investigate further the role of scaffold proteins in mediating the phenomena we report.

In contrast to the BRAF-selective inhibitors, the pan-RAF inhibitors appear to induce paradoxical activation of CRAF. They induce BRAF binding to CRAF and CRAF activation, but do not activate MEK–ERK signaling. We posit that this is because these agents target both BRAF and CRAF. Thus, although their inhibition of BRAF will stimulate CRAF activation, they will simultaneously inhibit CRAF (Figure 7C). This model is supported by our observation that ^T421N^CRAF converts sorafenib from a pathway inhibitor to a pathway activator and we argue that the paradoxical activation of CRAF by these inhibitors is mediated by BRAF, rather than disrupted feedback inhibition as previously suggested (Hall-Jackson et al., 1999).

Recently, paradoxical activation of PKB/AKT and PKCɛ was also described (Cameron et al., 2009; Okuzumi et al., 2009). While ATP-competitive inhibition can block kinase function, they do not block the upstream events that activate the target kinase. For instance, PKB/AKT inhibitors block the function of this kinase, but occupation of the ATP-pocket by these inhibitors was sufficient to induce the priming phosphorylation usually required for its full activation (Okuzumi et al., 2009). Inhibitor binding to PKCɛ has been shown to have a similar effect (Cameron et al., 2009). Importantly, the paradoxical activation of PKB/AKT and PKCɛ did not result in pathway activation because of the continued presence of the inhibitors (Frye and Johnson, 2009). In contrast, although BRAF inhibitors also block BRAF kinase activity, this relieves auto-inhibition and results in BRAF hyperphosphorylation, BRAF binding to CRAF, pathway activation and oncogenesis, all presumably because BRAF can heterodimerize with CRAF. Our study also highlights the critical difference between BRAF-selective and pan-RAF drugs. Whereas BRAF-selective drugs cause pathway activation in a RAS-dependent manner, this does not occur with pan-RAF drugs.

Our results provide important insight into the genetics of human cancer. Excluding V600 mutants, D594 mutants are the third most common in BRAF in cancer (34 out of 443 cases or ∼7.7%; www.sanger.ac.uk/genetics/CGP/cosmic/). Furthermore, as mentioned in the Introduction, while *BRAF* and *RAS* mutations are generally mutually exclusive, 4 of the 34 (11.8%) tumors with D594 mutations also have mutations in RAS. This is a highly significant enrichment for the coincidence of these mutations (p < 10^−9^; Fisher's Exact Test) and suggests a functional interaction. We now provide strong circumstantial evidence of such an interaction using transgenic mice. By themselves, ^D594A^Braf and ^G12D^Kras do not induce melanoma, but they cooperate to induce rapid onset melanoma. This highly significant result (p < 0.0002) provides a rational explanation for the coincidence of these mutations in human cancer. Furthermore, we show that the BRAF inhibitors also hyperactivate this pathway in growth factor stimulated cells, providing an explanation of why kinase dead BRAF mutations are not always coincident with RAS mutations; presumably in some tumors the cooperating mutation is upstream of RAS.

Our results also suggest several potential mechanisms by which resistance to RAF targeting drugs could develop in patients. BRAF mutant tumors could become resistant to BRAF-selective drugs, if they acquire a mutation in RAS or an upstream component that activates RAS, or if the drugs select a population of cells harboring pre-existing mutations in RAS. Theoretically this would cause BRAF-mediated CRAF activation, which may not only induce resistance, but could potentially promote tumor growth. In line with this, increased expression of CRAF can mediate acquired resistance to pan-RAF drugs in BRAF mutant cancer cells in vitro (Montagut et al., 2008), establishing that CRAF can mediate resistance under some circumstances. Our in vitro studies also suggest that a potential mechanism of resistance in patients with RAS mutant tumors being treated with pan-RAF drugs is acquisition (or selection for cells with pre-existing mutations) of a CRAF mutation such as a gatekeeper mutant that prevents drug binding. Again this would potentially result in BRAF-mediated activation of CRAF (Figure 7D) and possibly accelerated tumor growth.

Although our studies are restricted to cell lines and transgenic mice, they do have important immediate clinical implications. They strongly argue that BRAF-selective inhibitors should not be administered to patients with RAS mutant tumors, because long-term use could accelerate tumor growth. Intriguingly, 10%–15% of patients treated with BRAF-selective drugs develop squamous cell carcinoma (SCC)(Flaherty et al., 2009; Schwartz et al., 2009). Although MEK–ERK signaling has not yet been implicated in this response, 22% of SCCs harbour oncogenic mutations in *RAS* (9% *HRAS*, 8% *NRAS*, 5% *KRAS*: www.sanger.ac.uk/genetics/CGP/cosmic/), raising the intriguing possibility that the BRAF-selective drugs act as tumor promoters in premalignant skin cells harboring existing mutations in RAS and/or activation of upstream components that activate RAS.

While sorafenib is equipotent for wild-type and ^V600E^BRAF (Wilhelm et al., 2004), the BRAF inhibitors we used are approximately 10-fold more active against ^V600E^BRAF (King et al., 2006; Tsai et al., 2008). Nevertheless, our data establish that they target wild-type BRAF in RAS mutant cells. The problem of mutant v.s. wild-type protein specificity is likely to be difficult to resolve, because whereas full inhibition of ^V600E^BRAF may be necessary for clinical response in BRAF mutant tumors, activation of only a small proportion of wild-type BRAF could be sufficient to activate the pathway in RAS mutant cells. Thus, to achieve efficacy against ^V600E^BRAF but avoid activation of wild-type BRAF in RAS mutant cells, the drugs will need to be exquisitely selective for the mutant protein. Alternatively, pan-RAF drugs may be effective because they will target both ^V600E^BRAF and CRAF activated by BRAF in RAS mutant tumors. Furthermore, our data suggest that CRAF or MEK selective drugs should be used in RAS mutant tumors, because they do not induce BRAF-CRAF complexes and will not activate the pathway if the tumors acquire mutations such as ^T421N^CRAF that block drug binding. Perhaps RAF and MEK inhibitors should be combined to provide the best responses and prevent emergence of resistance, but these issues need to be balanced against the urgency of the clinical problem being addressed.

In summary, we show that inhibition of BRAF in RAS mutant cancer cells leads to MEK hyperactivation through CRAF. We have elucidated another mechanism by which BRAF activates MEK–ERK signaling, not only to drive tumorigenesis and tumor progression, but also potentially to allow development of de novo or acquired resistance to RAF-targeted therapies. Clearly, BRAF is a remarkably versatile oncogene that can promote MEK–ERK activation and tumor progression through several mechanisms and these will require different therapeutic strategies for effective disease management. Notably, many of the mutations that occur in other kinases in cancer are also predicted to cause inactivation (www.sanger.ac.uk/genetics/CGP/cosmic/). Our data raise the possibility that these could also act as idiosyncratic gain-of-function mutations that drive tumorigenesis. This study also raises important clinical questions and highlights the importance of fully understanding how signaling networks function to fully comprehend how patients may respond to targeted drugs. They also highlight the importance of genetic screening for patients, not only to identify those who are likely to respond, but to exclude those who could experience adverse effects and thereby ensure successful implementation of personalized medicine.

## Experimental Procedures

### Reagents

Expression vectors for epitope-tagged BRAF and CRAF have been described (Wan et al., 2004). For western blotting the following antibodies were used: rabbit anti-ppMEK1/2 and mouse anti-myc 9B11 (Cell Signaling Technology); mouse anti-NRAS (C-20), rabbit anti-ERK2 (C-14), rabbit anti-ARAF (C-20), mouse anti-BRAF (F-7) (Santa Cruz Biotechnology); mouse anti-Tubulin, and mouse anti-ppERK1/2 (Sigma); mouse anti-CRAF (for western blotting) (BD Transduction Laboratories). For immunoprecipitation, the following antibodies were used: rabbit anti-myc (Abcam); rabbit anti-CRAF (C-20;Santa Cruz Biotechnology); mouse anti-BRAF (F-7) (Ab from Santa Cruz Biotechnology). Calf intestinal phosphatase (CIP) was from New England Biolabs (NEB). PD184352, sorafenib and PLX4720 were synthesized in-house; 885-A was synthesized by Evotec AG (Abingdon, UK). All drugs were prepared in DMSO. Synthetic routes are available on request.

### Cell Culture Techniques

Human cell lines were cultured in DMEM (A375, WM852, HCT116, SW620, and PMWK) or RPMI (D04, MM485, MM415, and WM1791c) supplemented with 10% fetal bovine serum. For protein depletion, 3 × 10^5^ D04 cells were transfected with 5nM CRAF (5′-AAGCACGCTTAGATTGGAATA-3′) or NRAS (5′-CATGGCACTGTACTCTTCTCG-3′) specific, or scrambled control (5′-AAACCGTCGATTTCACCCGGG-3′) siRNA using INTERFERin as recommended by the manufacturer (Polyplus Transfection SA). For transient expression studies, D04 cells were transfected using the Amaxa Nucleofector System as recommended by the manufacturer (Lonza). COS-7 cells were propagated, transfected, and extracted as described (Wan et al., 2004). For generation of stable lines, D04 cells were transfected with pMCEF-FLAG-CRAF or pMCEF-FLAG-^T421N^CRAF using Effectene as recommended by the manufacturer (Invitrogen) and selected in G418 (1 mg/ml).

Cell lysates were prepared with NP40 buffer as described (Wan et al., 2004). For immunoprecipitation, lysates were incubated with 2 μg BRAF F-7, 5 μg CRAF C-20 or 2 μg rabbit anti-myc antibodies, captured on Protein G sepharose 4B beads (Sigma) and analyzed by western blotting using standard protocols. Specific bands were detected using fluorescent-labeled secondary antibodies (Invitrogen; Li-COR Biosciences) and analyzed using an Odyssey Infrared Scanner (Li-COR Biosciences). For CIP treatment, immunoprecipitates were washed twice with NP40 lysis buffer, once in CIP buffer (50 mM Tris-Cl [pH 7.5], 150 mM NaCl, 10 mM MgCl_2_, and 1 mM EDTA), and incubated with CIP with or without 0.2 mM Na_3_VO_4_ and 7 mM EDTA. The immunoprecipitates were washed in CIP buffer and western blotted. Coupled RAF kinase assays were performed with immunoprecipitated CRAF or BRAF as described (Wan et al., 2004). Membrane fractionation was as described (Garnett et al., 2005).

### Transgenic Mice

Experiments were performed under Home Office license authority in accordance with United Kingdom Coordinating Committee on Cancer Research Guidelines (Workman et al., 1988) and with local Ethics Committee approval. To activate CreERT2, mice were treated with four doses (10mg each) of topically applied tamoxifen as described (Dhomen et al., 2009). Genotyping was performed by PCR. *Braf^LSL-D594A^* and *Braf^Lox-D594A^* was analyzed as described for *Braf^LSL-V600E^* and *Braf^Lox-V600E^* respectively and *Tyr::CreERT2* was analyzed as described (Dhomen et al., 2009). *Kras^LSL-G12D^* was analyzed using primers 5′-CGCAGACTGTAGAGCAGCG-3′ and 5′-CCATGGCTTGAGTAAGTCTGC-3′. For expression analysis, RNA was prepared (QIAGEN RNEasy, QIAGEN) and first-strand cDNA synthesis was performed with 500ng total RNA and random hexanucleotides (Random Primers, Invitrogen). Specific genes were amplified under linear conditions for analysis as described (Dhomen et al., 2009). For *Kras* cDNA sequencing, a 238 bp fragment of Kras cDNA was PCR amplified using primers 5′-GGCGGCAGCGCTGTGGCGGCG-3′ and 5′-CGTAGGGTCATACTCATCCAC-3′ and sequenced using automated dideoxy sequencing.

For immunohistochemistry (IHC), tissues were fixed and analyzed as described (Dhomen et al., 2009). Positive (a well characterized sample of mouse melanoma) and negative (omission of the primary antibody and substitution with preimmune serum) controls were included in each slide run. Immunohistochemical staining was analyzed by two of the authors on a multi-headed microscope. Tumor cell lines were established by mechanically dissociating tumors in DMEM/20%FCS/Primocin (0.1mg/ml - InvivoGen) and clonal lines were selected by limiting dilution.

**Extended Experimental Procedures**

**Reagents**

For western blotting the following antibodies were used: rabbit anti-ppMEK1/2 and mouse anti-myc 9B11 (Cell Signaling Technology); mouse anti-NRAS (C-20), rabbit anti-ERK2 (C-14), rabbit anti-ARAF (C-20), mouse anti-BRAF (F-7) (Santa Cruz Biotechnology); mouse anti-Tubulin, and mouse anti-ppERK1/2 (Sigma); mouse anti-CRAF (for western blotting) (BD Transduction Laboratories). For immunoprecipitation, the following antibodies were used: rabbit anti-myc (Abcam); rabbit anti-CRAF (C-20; Santa Cruz Biotechnology); mouse anti-BRAF (F-7) (Santa Cruz Biotechnology). Calf intestinal phosphatase (CIP) was from New England Biolabs (NEB). PD184352, sorafenib and PLX4720 were synthesized in-house; 885-A was synthesized by Evotec AG (Abingdon, UK). All drugs were prepared in DMSO. Synthetic routes are available on request.

**Expression Constructs**

The expression vectors for wild-type human CRAF and wild-type human BRAF, pEFm/CRAF and pEFm/BRAF respectively have been described (Marais et al., 1995). Briefly, the vector backbone is pUC19 and the elongation factor 1α (EF1α) promoter is used to drive exogenous protein expression. The vector includes the first intron from human EF1α to assist mRNA processing during expression. The β-globin 5′ and 3′ untranslated regions (UTRs) are used to provide a strong translation start site (5′ UTR), and also to provide mRNA stability and a poly adenylation signal (3′ UTR). The vector introduces an amino-terminal myc-epitope tag (EQKLISEEDL) onto the exogenously expressed protein. The BRAF coding region includes the alternatively spliced exons 1 and 2 but not exons 8b or 10a and various modifications were introduced to provide additional restriction sites (without changing the amino acid sequence) and alterations to the 3′-UTR to allow easier manipulation of this construct. Standard PCR-directed mutagenesis approaches were used to generate the various mutations used in the study and all mutations were verified by automated dideoxy sequencing. The expression vector pMCEF/FLAG/CRAF uses the same expression cassette, but the backbone also possesses a neo resistance cassette to facilitate selection in the presence of G418. In addition, a version of this vector was used that incorporates a FLAG (DYKDDDKGS), rather than a myc-epitope tag.

**Cell Culture**

Human cell lines were cultured in DMEM (A375, WM852, HCT116, SW620, PMWK, SKMel24, SKMel28, D25) or RPMI (D04, MM485, MM415, WM1791c) supplemented with 10% fetal bovine serum. African green monkey kidney COS-7 cells were cultured in DMEM supplemented with 10% fetal bovine serum. All cell lines were incubated at 37°C and 10% CO_2_. For inhibitor treatment, the drugs were dissolved in DMSO and added to the medium for 2-5 hr. When two compounds were used, the first was added 30 min prior to the second. For cell growth assays, cells were seeded in 96-well plates and treated with drug in quadruplicate in a 10-point titration assay for 5 days. The amount of growth (% DMSO controls) was determined using sulphorhodamine B reagent (Monks et al., 1991) as follows. 1,000-10,000 cells (depending on cell type) were plated into 96-well plates in 100 μL medium. After 24 hr, compounds prepared in DMSO (10mM stocks) were serially diluted in culture medium at 2× the final required concentrations and 100μL were added to the cells to nine final concentrations of 0.005 μM-100 μM. After a further 5 days, the cells are fixed in trichloroacteic acid (10%), and stained with sulforhodamine-B (0.1%). After rinsing, the bound stain was disolved using 100 μL 10 mM Tris (pH 8.0) and the absorbance at 540 nm determined. The data were analyzed by nonlinear regression to a four-parameter logistic equation (Graphpad Prism, Graphpad Software Inc., San Diego, CA, USA) and the GI_50_ value determined.

**siRNA Transfections**

3 × 10^5^ D04 cells per 35 mm diameter well were seeded in 2ml growth medium the day before transfection. The cells were either mock-transfected or transfected with 6nM CRAF-specific (5′-AAGCACGCTTAGATTGGAATA-3′) or NRAS-specific (5′-CATGGCACTGTACTCTTCTCG-3′) siRNA using INTERFERin as recommended by the manufacturer (Polyplus Transfection SA). Briefly, 0.6 μl of 20 μM siRNA and 6 μl of INTERFERin were combined in a total of 200 μl serum free medium in RNase-free tubes. The mix was vortexed for 10 s and incubated for 5-10 min before adding the complexes dropwise to the cells. The cells were serum-starved the day after transfecting and extracts were prepared 48 hr after transfection.

**DNA Transfections**

For transient protein expression in D04 cells, Lonza Nucleofector Technology (Lonza, Cologne AG) was used. 2 μg of DNA was mixed with 1x10^6^ cells resuspended in 100 μl of Nucleofection Solution V in an Amaxa-certified cuvette and transfected using program T030. The cells were re-plated into 35mm diameter tissue culture wells and incubated for 48 hr before preparation of cell extracts.

For generation of stable lines, D04 cells were transfected using Effectene (Invitrogen) and selected in G418. 3-4x10^5^ cells were plated in 35 mm diameter wells and incubated overnight. 0.4 μg of DNA diluted into 100 μl of DNA condensation buffer (EC) and 3.2 μl enhancer were mixed vigorously and incubated for 2-5 min. 10 μl Effectene reagent was added and the mixture was incubated for another 5-10 min. The cells were washed with 2ml PBS and 1.6ml fresh serum containing medium was added. The DNA complexes were diluted with 600 μl of culture medium and the mixture added to the cells drop-wise. After six hours, the medium was replaced with 2ml of fresh growth medium. After 48 hr, the cells were replated into several 10cm dishes in a 10-fold dilution series and incubated in G418 (1mg/ml) for selection. The medium was refreshed weekly and after 2-3 weeks, single colonies were selected and expanded.

For transient expression in COS-7 cells, Lipofectamine (Invitrogen) was used. 2x10^5^ cells were plated into 35mm diameter wells and incubated overnight. 75 to 200 ng of expression plasmid (depending on construct) was mixed with empty vector to a total of 700 ng DNA in 16μl PBS. Typically, 3 μl of Lipofectamine in 13 μl of PBS was added to the DNA on the surface of a bacterial plate and incubated (Lipofectamine is inactivated by binding to polypropylene) for 15 min at room temperature. The cells were washed twice with 1ml serum-free DMEM, and then overlaid with 800 μl of serum free DMEM. 200 μl of serum free DMEM was added to the DNA:Lipofectamine mix, and the total volume was added to the cells. After six hours, the complexes were removed and replaced with 2ml of normal culture medium. Cell extracts were prepared two days following transfection.

**Preparation of Cell Lysates**

Culture medium was aspirated from cells and cells were placed on ice and washed three times in ice-cold PBS. Depending on the assays, the cells were scraped into 50-200 μl Nonidet P40 (NP40) extraction buffer (Table S3) and incubated on ice for five minutes. The cells were sheared by passing through a pipette tip several times and the samples were centrifuged at 20,000 × *g* for 5 min at 4°C and the soluble fraction was harvested.

**RAF Coimmunoprecipitations**

Immunoprecipitations were performed in 300 μl cell lysates from one 35mm diameter well for endogenous protein or from 2-3 wells for transfected protein. Endogenous BRAF or CRAF were immunoprecipitated with 2μg BRAF F-7 or 5μg CRAF C-20 respectively and myc-tagged BRAF and CRAF with 2 μg rabbit anti-myc antibody. The antibody-protein complex was captured using 20 μl of a 1:1 Protein G sepharose 4B beads (Sigma-Aldrich) mixture in NP40 lysis buffer (Table S3) and immunoprecipitates (IPs) were mixed for 2 hr at 4°C on a rotation wheel. Thereafter, the IPs were washed three times with 300 μl of NP40 lysis buffer (Table S3) before analysis on standard sodium dodecyl sulfate polyacrylamide gel electrophoresis (SDS-PAGE). Specific bands were detected using fluorescent-labeled secondary antibodies (Invitrogen; Li-COR Biosciences) and analyzed using an Odyssey Infrared Scanner (Li-COR Biosciences). For CIP treatment, immunoprecipitates were washed twice with NP40 lysis buffer (Table S3) and once in CIP buffer (Table S3). Thereafter immunoprecipitates were incubated with 30 μl CIP buffer containing 5 units of CIP in the presence or absence of 0.2 mM Na_3_VO_4_ phosphatase inhibitor and 7mM EDTA. Controls were incubated in CIP buffer without CIP. The reactions were performed at 30°C for 30 min before analysis on SDS-PAGE.

**Cell Fractionation Experiments**

D04 cells were plated on two 10cm dishes per treatment and grown to confluency. After treatment, cells were washed three times with cold PBS, washed once with 20mM HEPES pH 7.4 and then lysed by scraping in 20mM HEPES pH 7.4 supplemented with protease inhibitors (1ml per 2 plates). Cells were disrupted by passing them through a 9G syringe ten times, followed by another ten times through a 19G syringe (Terumo Medical). Lysates were centrifuged at 900 × *g* for five minutes to pellet the nuclear proteins. The supernatant was transferred to fresh 1.5ml ultracentrifuge tubes (Beckman Coulter) and 200 μl removed as a total lysate control. The remainder was centrifuged at 100,000 × *g* for 30 min at 4°C to separate the cytosolic fraction from the membrane fraction. The supernatant containing the cytosolic fraction was transferred to a fresh 1.5ml tube and the pelleted membrane fraction washed once in 20 mM HEPES pH 7.4 before resuspension in 200 μl 20mM HEPES pH 7.4/1% Triton X-100. For analysis on SDS-PAGE, the concentration of protein was determined by Bradford protein assay (Bio-Rad Laboratories) using purified bovine serum albumin (BSA) as a standard as described by the manufacturer. Equal amounts of protein were loaded for the cytosolic fraction and total cell lysate. Three times as much protein was loaded for the membrane fraction.

**RAF Kinase Assays**

The in vitro kinase activity of endogenous RAF proteins or myc-tagged RAF proteins transiently expressed in COS-7 cells was measured using a coupled kinase cascade assay with GST-MEK, GST-ERK and myelin basic protein (MBP) (Sigma-Aldrich) as sequential substrates. ERK activation was quantified by measuring the incorporation of [^32^P]-orthophosphate (PerkinElmer) into MBP. For measurement of endogenous BRAF kinase activity, D04 or A375 cells were seeded in 10cm dishes and harvested in 300 μl of NP40 buffer (Table S3) as described above. Protein concentrations were determined and equal amounts of protein were immunoprecipitated as described above.

For measurement of mutant BRAF kinase activities, COS-7 cells were transiently transfected with myc-tagged BRAF and cells in one 35 mm diameter well were harvested in 200μl of NP40 buffer (Table S3). The relative concentrations of exogenously expressed RAF in these cell lysates were determined by quantitative western blotting using the myc antibody (Cell Signaling Technology) specified above. Bands were quantified using the Odyssey infrared imaging system (LI-COR Biosciences). Equivalent amounts of RAF were immunoprecipitated using rabbit myc antibody (Abcam) as specified above.

Endogenous and transiently transfected RAF proteins were immunoprecipitated in a total of 300 μl NP40 buffer (Table S3) for 2 hr at 4°C and immunoprecipitates were washed sequentially three times with chilled wash buffer (Table S3) containing decreasing concentrations of KCl (1M KCl, 0.1M KCl and no KCl). The first-step reaction was initiated by addition of 20 μl MKK buffer (containing GST-MEK and GST-ERK, Table S3) to the beads and incubated at 30°C for 10 min in the case of myc-tagged BRAF or 30 min for endogenous BRAF and CRAF. Reactions were terminated by the addition of 20 μl KILL buffer (Table S3), which contains EDTA to chelate Mg^2+^ ions and inhibit kinase activity. The reaction supernatants were collected from the beads and transferred into fresh tubes for the second step reaction. 5 μl of supernatant was incubated with 25 μl MBP buffer containing [γ-^32^P]ATP (PerkinElmer) for ten minutes at 30°C in triplicate to measure ERK activity. The second reaction was terminated by spotting 20 μl of reaction mix onto a 1cm^2^ piece of P81 paper (VWR International), which was then dropped into 400ml 25mM orthophosphate solution. The papers were washed three times in 400 ml 25 mM orthophosphate solution to remove the unincorporated ATP and the [^32^P]-orthophosphate incorporated into MBP was determined using Cerenkov counting. For transfected samples, the background counts were determined using lysates of cells transfected with the empty vector. For endogenous protein, samples in which no RAF was immunoprecipitated were used. Background values were removed and to ensure linearity, assays were used at below 50% saturation. To determine BRAF^WT^ and BRAF^T529N^ sensitivity to 885-A, immunoprecipitated BRAF was preincubated with drug in KCl-free buffer for 10 min at room temperature prior to the first-step reaction.

To measure the activity of purified ^V600E^BRAF, a 96-well DELFIA-based assay system was used. Full-length rabbit MEK1 protein was expressed with a GST tag at the N-terminus and a C-terminal histidine tag in *Escherichia coli* JM109 bacteria and purified by nickel-agarose affinity chromatography. Full length BRAF protein was generated by infection of SF9 insect cells with a recombinant baculovirus expressing full-length human ^V600E^BRAF with an N-terminal histidine tag and purified as above. For the kinase assays, all incubations were at room temperature with shaking. 4 μg GST-MEK1, 100-200ng purified ^V600E^BRAF and 1 μl inhibitor at the required concentrations (0.001 to 100 μM final concentration) were added to the wells of glutathione-coated plates and preincubated for 10 min. ATP in DELFIA assay buffer (20 μL; Table S3), to give a final concentration of 100 μM, was added to each well, and the plates were incubated for 45 min. The plates were washed 3X with 200 μl 0.1% tween20/water. Primary antibody (rabbit anti-phospho MEK1/2 diluted 1/2000, Cell Signaling Technologies) and Eu-labeled anti-rabbit secondary antibody (diluted 1/1000, Perkin-Elmer) were preincubated for 30 min and 100 μl was added to the plates and incubated for a further hour. The plates were washed as before, and 100 μl DELFIA enhancement solution (Perkin-Elmer, Turku, Finland) was added. The plates were sealed and incubated for 30 min and europium counts measured on Spectramax M5 plate reader (Molecular Devices, Wokingham, UK). IC_50_ values were determined using GraphPad Prism (Graphpad Software Inc., San Diego, CA, USA).

**Transgenic Mice**

Experiments were performed under Home Office license authority with local Ethics Committee approval. To activate CreERT2, four doses of tamoxifen (Sigma; 10mg each in 100% ethanol) were applied topically to the shaven skin on the backs of the mice every other day for 7 days. Genotyping was performed by PCR using DNA prepared with DNeasy kits (QIAGEN). *Braf^LSL-D594A^* and *Braf^Lox-D594A^* were analyzed using primers: A) 5′-GCCCAGGCTCTTTATGAGAA-3; B) 5′-AGTCAATCATCCACAGAGACCT-3′; and C) 5′-GCTTGGCTGGACGTAAACTC-3′. A+B detects the wild-type BRAF allele (466 bp product) and *Braf^Lox-D594A^*, the Cre-recombinase recombined allele (518pb product). A+C detects the targeted allele *Braf^LSL-D594A^* (140 bp). *Tyr::CreERT2* was analyzed using primers 5′-GAAGCAACTCATCGATTG-3′ and 5′-TGAAGGGTCTGGTAGGATCA-3′. *Kras^LSL-G12D^* was analyzed using primers 5′-CGCAGACTGTAGAGCAGCG-3′ and 5′-CCATGGCTTGAGTAAGTCTGC-3′.

mRNA expression analysis was performed by RT-PCR. RNA was prepared using the RNEasy kit (QIAGEN). First-strand cDNA synthesis was performed with 500ng total RNA and random hexanucleotides (Random Primers, Invitrogen). Tyrosinase (Tyr), was detected using primers 5′-TGGTTCCTTTCATACCGCTC-3′ and 5′-CAGATACGACTGGCTTGTTCC-3′; Dct with 5′-GCAAGATTGCCTGTCTCTCC-3′ and 5′-AGTCCAGTGTTCCGTCTGCT-3′; Pax3 with 5′-CCAGGATGATGCGGCCCGGCCCGGG-3′ and 5′-AGGATGCGGCTGATAGAACTCACTG-3′; and silver/gp100 (Si) with 5′-GGAGAGGTGGCCAGGTATC-3′ and 5′-CAGTAATGGTGAAGGTTGAAC-3′. The control Gapdh was detected with 5′- GATGGCCCCTCGGAAAGCT-3′ 5′-CCAGTGAGCTTCCCGTTCAGC-3′. To sequence *Kras* cDNA, a 238 bp product from Kras cDNA was PCR amplified using primers 5′-GGCGGCAGCGCTGTGGCGGCG-3′ and 5′-CGTAGGGTCATACTCATCCAC-3′ and directly sequenced using these primers and automated dideoxy sequencing.

For immunohistochemistry (IHC), tumors were fixed in 10% buffered formalin and embedded in paraffin. Sections (3-10μm) were stained with hematoxylin and eosin using standard protocols. For S100 and Ki67 staining, antigen retrieval was performed in citrate buffer (pH 6.0, 30 min) and revealed using a rabbit polyclonal antibody (Dako, 1/1000), the Rabbit Envision Peroxidase kit and the AEC substrate chromogen (Dako) for S100, and a rat monoclonal antibody (Dako,1/25), the rat Vectastain ABC kit (Vector Labs, USA) and DAB as chromagen for Ki67. Positive (a well characterized sample of mouse melanoma) and negative (omission of the primary antibody and substitution with preimmune serum) controls were included in each slide run. Immunohistochemical staining was analyzed by two of the authors on a multi-headed microscope. Tumor cell lines were established by mechanically dissociating tumors in DMEM/20%FCS/Primocin (0.1mg/ml - InvivoGen) and clonal lines were selected by limiting dilution.

## Supplemental Information

Document S1. Three Tables

Document S2. Article Plus Supplemental Information

## Footnotes

Supplemental Information includes Extended Experimental Procedures, four figures, and three tables and can be found with this article online at doi:10.1016/j.cell.2009.12.040.
