# Supplementary material for: Kinase-Dead BRAF and Oncogenic RAS Cooperate to Drive Tumor Progression through CRAF
Source: Cell. 2010 Jan 22;140(2):209–21. doi: 10.1016/j.cell.2009.12.040 (PMC2872605; doi:10.1016/j.cell.2009.12.040)
Supplement: Document S1. Three Tables [file mmc1.pdf]

Kinase	% Remaining Activity	Kinase	% Remaining Activity
MKK1	78	CHK1	86
ERK1	100	CHK2	71
ERK2	77	GSK3b	99
JNK1	95	CDK2-Cyclin A	104
JNK2	80	PLK1	92
p38a MAPK	87	PLK1 (Okadaic Acid)	96
P38b MAPK	71	AURORA B	93
p38g MAPK	99	AMPK	90
p38s MAPK	87	MARK3	104
ERK8	86	BRSK2	95
RSK1	82	MELK	83
RSK2	74	CK1	39
PDK1	98	CK2	96
PKBa	93	DYRK1A	103
PKBb	88	DYRK2	102
SGK1	108	DYRK3	87
S6K1	96	NEK2a	77
PKA	92	NEK6	77
ROCK 2	77	IKKb	80
PRK2	84	PIM1	81
PKCa	106	PIM2	89
PKC zeta	85	PIM3	72
PKD1	85	SRPK1	107
MSK1	73	MST2	87
MNK1	77	EFK2	98
MNK2	96	HIPK2	73
MAPKAP-K2	104	PAK4	82
PRAK	86	PAK5	81
CAMKKb	96	PAK6	87
CAMK1	87	Src	68
SmMLCK	70	Lck	58
PHK	77	CSK	92

Table S1. 885-A is inactive against a panel of other kinases in vitro.

Inhibition profiling of 885-A against a panel of 64 kinases at a fixed concentration of 1μM. Values are the % remaining activity compared to DMSO-treated controls.

Cell Line	Tumour type	BRAF	KRAS	NRAS
A375	Melanoma	V600E	WT	WT
D24	Melanoma	V600E	WT	WT
SkMel24	Melanoma	V600E	WT	WT
SkMEl28	Melanoma	V600E	WT	WT
WM266.4	Melanoma	V600D	WT	WT
D04	Melanoma	WT	WT	NRAS Q61L
MM415	Melanoma	WT	WT	NRAS Q61L
MM485	Melanoma	WT	WT	NRAS Q61R
WM852	Melanoma	WT	WT	NRAS Q61R
PMWK	Melanoma	WT	WT	WT
WM1791c	Melanoma	WT	T50I/Q61H	WT
HCT116	colorectal carcinoma	WT	G13D	WT
SW620	colorectal carcinoma	WT	G12V	WT

Table S2. Cell lines used in this study

The cell lines used in this study are shown, together with the mutations that they harbor in RAS or BRAF.

Buffer	Composition
**NP40 protein extraction**	50mM Tris-Cl pH 7.5, 150mM NaCl, 0.5% (v/v) Igepal, 5mM NaF, 0.2mM Na_3VO_4, 5μg/ml aprotinin, 5μg/ml leupeptin
**CIP**	50mM Tris-Cl pH 7.5, 150mM NaCl, 10mM $MgCl_2$, 1mM EDTA
**WASH**	30mM Tris-Cl pH 7.5, 0.2mM EDTA, 0.3% (v/v) β-mercaptoethanol, 10% (v/v) glycerol, 0.1% (v/v) Triton X-100, 5mM NaF, 0.2mM Na_3VO_4, 1M/0.1M/no KCl
**MKK**	30mM Tris-Cl pH 7.5, 0.1mM EDTA, 10mM $MgCl_2$, 0.1% (v/v) Triton X-100, 5mM NaF, 0.2mM Na_3VO_4, 0.3% (v/v) β-ME, 6.5μg/ml (~0.093 μM) GST-MEK, 400μg/ml (~6.68 μM) GST-ERK, 5mM ATP
**KILL**	30mM Tris-Cl pH 7.5, 9.0mM EDTA, 0.1% (v/v) Triton X-100, 5mM NaF, 0.2mM Na_3VO_4, 0.3% (v/v) β-ME
**MBP**	50mM Tris-Cl pH 7.5, 0.1mM EDTA, 12mM $MgCl_2$, 0.1% (v/v) Triton X-100, 5mM NaF, 0.2mM Na_3VO_4, 0.3% (v/v) β-ME, 200μg/ml BSA, 1mg/ml MBP, 0.12MBq [γ^{32}P]-ATP (5.55 x 10^8MBq/mmol; PerkinElmer)
**DELPHIA ASSAY BUFFER**	20mM MOPS, pH 7.2, containing 5mM EGTA, 10mM $MgCl_2$, 0.1% β-ME, 25mM β-glycerophosphate

Table S3. Composition of buffers used in this study
